# Mosaic genome-wide maternal isodiploidy: an extreme form of imprinting disorder presenting as prenatal diagnostic challenge

**DOI:** 10.1186/s13148-017-0410-y

**Published:** 2017-10-13

**Authors:** Susanne Bens, Manuel Luedeke, Tanja Richter, Melanie Graf, Julia Kolarova, Gotthold Barbi, Krisztian Lato, Thomas F. Barth, Reiner Siebert

**Affiliations:** 1 Institute of Human Genetics, University of Ulm & Ulm University Hospital, Albert-Einstein-Allee 11, D-89081 Ulm, Germany; 2 Department of Obstetrics and Gynecology, University of Ulm & Ulm University Hospital, Ulm, Germany; 3 Institute of Pathology, University of Ulm & Ulm University Hospital, Ulm, Germany

**Keywords:** Genome-wide maternal uniparental disomy, Imprinting, Prenatal diagnostics, DNA methylation, Multilocus imprinting disturbances

## Abstract

**Background:**

Uniparental disomy of certain chromosomes are associated with a group of well-known genetic syndromes referred to as imprinting disorders. However, the extreme form of uniparental disomy affecting the whole genome is usually not compatible with life, with the exception of very rare cases of patients with mosaic genome-wide uniparental disomy reported in the literature.

**Results:**

We here report on a fetus with intrauterine growth retardation and malformations observed on prenatal ultrasound leading to invasive prenatal testing. By cytogenetic (conventional karyotyping), molecular cytogenetic (QF-PCR, FISH, array), and methylation (MS-MLPA) analyses of amniotic fluid, we detected mosaicism for one cell line with genome-wide maternal uniparental disomy and a second diploid cell line of biparental inheritance with trisomy X due to paternal isodisomy X. As expected for this constellation, we observed DNA methylation changes at all imprinted loci investigated.

**Conclusions:**

This report adds new information on phenotypic outcome of mosaic genome-wide maternal uniparental disomy leading to an extreme form of multilocus imprinting disturbance. Moreover, the findings highlight the technical challenges of detecting these rare chromosome disorders prenatally.

**Electronic supplementary material:**

The online version of this article (10.1186/s13148-017-0410-y) contains supplementary material, which is available to authorized users.

## Background

Uniparental disomy (UPD) refers to the constellation of two identical (isodisomy) or homologous (heterodisomy) chromosomes inherited from only one parent [1]. Known mechanisms resulting in UPD are gametic complementation, monosomy or trisomy rescue, compensatory UPD, and post-fertilization errors including mitotic recombination [2]. Non-disjunction at meiosis I can result in heterodisomy and isodisomy while non-disjunction at meiosis II leads to isodisomy of those parts of the chromosome set not involved in homologous recombination [1, 3]. Clinical consequences of isodisomy include unmasking of recessive diseases by transmitting two affected gene copies from one heterozygous parent carrier [2]. Moreover, UPD can cause imbalance of imprinted gene expression when the involved regions are subject to genomic imprinting, i.e., showing a parent-of-origin specific gene expression [2, 4]. In these cases, UPD results in the inheritance of either two active or two repressed copies of a gene, dependent on the chromosomal region involved and the sex of the transmitting parent [4]. Indeed, UPD is a well-known mechanism underlying several imprinting disorders including Prader-Willi syndrome [PWS, UPD(15)mat], Angelman syndrome [AS, UPD(15)pat], Beckwith-Wiedemann syndrome [BWS, UPD(11p15.5)pat], transient neonatal diabetes mellitus [TNDM, UPD(6)pat], Silver-Russell syndrome [SRS, UPD(7)mat, UPD(11p15.5)mat], Temple syndrome [TS14, UPD(14)mat], Kagami-Ogata syndrome [KOS14, UPD(14)pat], and pseudo-hypoparathyroidism type IB [PHP-1B, UPD(20)pat]. The frequency of UPD in the aforementioned imprinting syndromes varies and ranges from 1 to 2% (AS) [5] up to about 40% (TNDM) [6]. Apart from UPD, imprinting disorders can result from primary epimutation [7]. The latter term in this context refers to a change in DNA methylation at the differentially methylated region (DMR) regulating the parent-of-origin specific gene expression without evidence for a genomic mutation in *cis* [7]. In these instances, the DNA methylation disturbance can affect more than one locus and is then referred to as “multilocus imprinting disturbance” (MLID). To date, MLID has been reported for most of the phenotypes associated with the classical imprinting disorders except KOS14 and PWS [8–10]. However, the amount and combination of loci affected is highly heterogeneous and generally does not extend to all known imprinted regions.

The most extreme form of uniparental inheritance is uniparental diploidy, i.e., UPD of all chromosomes, leading to genome-wide DNA methylation disturbances at virtually all imprinting loci in a single patient. This global imbalance of imprinting seems to be not compatible with life, since the constellation is known to be lethal in mammals [11]. However, in a mosaic state, genome-wide UPD can lead to live-born children [4, 12]. So far, only about 18 cases of genome-wide UPD have been reported in the literature and only two of them show mosaic genome-wide UPD of maternal origin.

We here add one further case to the short list of patients with this extremely rare genetic disorder that we recently diagnosed by prenatal genetic testing. We provide a detailed description of the diagnostic work-up and phenotypic outcome. Our investigations include prenatal analyses of DNA methylation at major regulatory sites associated with the classical imprinting syndromes.

## Methods

### Case report

A 28-year-old pregnant woman (height 167 cm, weight prior to pregnancy 97 kg) and her 31-year-old partner, both healthy, non-consanguineous, and with uneventful family history, presented at our genetic department. During ultrasound examination in the 17 + 0 week of gestation in our center, the fetus showed intrauterine growth retardation, left diaphragmatic hernia with parts of stomach and bowel localized in the chest, dextrocardia, a short nasal bone, and single umbilical artery. These findings were confirmed at the 18 + 1 week when the pregnancy was terminated. The pregnancy was conceived spontaneously.

### Histopathology of placenta tissue

For histopathological examination, formalin-fixed and paraffin-embedded tissue obtained from termination of pregnancy was stained with hematoxylin and eosin according to standard protocols.

### Quantitative fluorescence-polymerase chain reaction (QF-PCR) for chromosomes 13, 18, 21, X, and Y

PCR was carried out using fluorescent-labeled primer in the PCR reaction. Primer sequences are given in Additional file 1. Fragment analysis was performed with a capillary sequencer according to standard procedures. Analyzed material included uncultured and cultured amniotic cells, placenta tissue, and peripheral blood of the couple. In all samples, the markers IFNAR, D21S11, D21S1270, D21S1437, D21S1446, and PentaD on chromosome 21; D13S742, D13S634, and D13S628 on chromosome 13; D18S391, D18S1002, D18S535, and D18S286 on chromosome 18; and HPRT, P39, DXS981, DXS6854, and DXS1283E on chromosome X as well as AMX/Y for gonosomal constellation were investigated.

### Conventional karyotyping

Cytogenetic banding analyses using GTG-banding according to standard techniques were performed on long-term cultured amniotic fluid cells as well as phytohaemagglutinin (PHA)-stimulated lymphocytes from peripheral blood of both parents cultured for 72 h.

### Fluorescence in situ hybridization (FISH) analyses

Fluorescence in situ hybridization analyses were performed on uncultured and cultured amniotic fluid cells and placental tissue following standard protocols. The AneuVysion-Kit (Abbott/Vysis, Illinois, USA) was used for analyses of chromosomes 13, 18, 21, X, and Y. The LSI IGH/MYC/CEP8 Tri-Color Dual Fusion Probe Kit from Abbott/Vysis was applied for analyses of chromosomes 8 and 14. Furthermore, a previously described probe for the *CCND2* locus localized in 12p13, containing the fluorescent-labeled BAC clones RP11-578L13 and RP11-388F6 [13] mixed with CEP10 localized on chromosome 10 (Abbott/Vysis), was applied. Evaluation of FISH was conducted according to standard procedures [14] using Zeiss fluorescence microscopes equipped with appropriate filter sets. Digital image acquisition and processing were performed using ISIS digital image analysis system (MetaSystems, Altlussheim, Germany).

### Array-based (OncoScan) analyses

DNA was extracted from uncultured and cultured amniotic fluid cells according to standard methods and hybridized on an OncoScan Array (Affymetrix, Santa Clara, CA, USA). Arrays were scanned and analyzed with the Chromosome Analysis Suite (ChAS) v3.1.0.15 and the OncoScan® Console 1.3 Software from Affymetrix as well as the Nexus Express software for OncoScan 3.1 (Bio Discovery, El Segundo, CA, USA). For analyses of allele ratios, the B allele frequency was exported with the Analysis Workflow tool of ChAS. For calculation of B allele frequencies (BAF), only informative parental homozygous markers were taken into account in which the fetus would be expected to show heterozygous calls [e.g., mother BB (BAF 1), father AA (BAF 0) or vice versa and expected child AB or BA (BAF 0.5)].

### Methylation-specific multiplex ligation-dependent probe amplification (MLPA)

For copy number and methylation analysis, the methylation-specific Salsa MS-MLPA Kit ME034-A1 (MRC Holland, Amsterdam, The Netherlands) was used according to the manufacturer’s instructions. This kit contains methylation-sensitive probes for the imprinted regions at the *PLAGL1*, *MEST*, *H19*, *KCNQ1OT1*, *MEG3*, *SNRPN*, *PEG3*, and *GNAS* (NESP55, NESPAS, GNASXL, GNAS) loci. Analyses and interpretation were performed with the commercially available software Sequence Pilot version 4.3.1 (JSI medical systems, Ettenheim, Germany). Three samples from amniocentesis with normal cytogenetic and QF-PCR results served as controls.

## Results

### Quantitative fluorescence-polymerase chain reaction (QF-PCR) for chromosomes 13, 18, 21, X, and Y

#### Uncultured amniotic cells

**Fig. 1:**
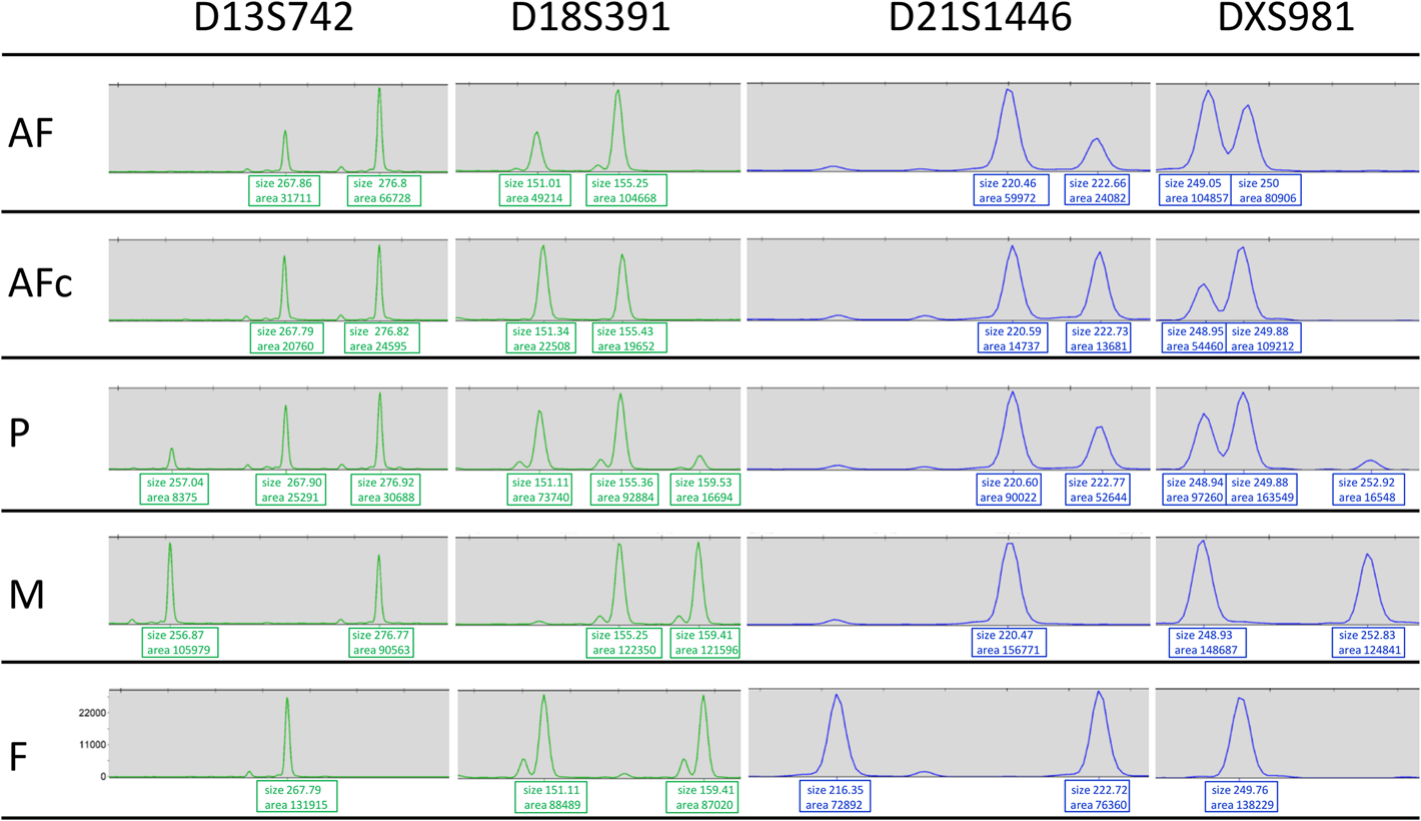
Microsatellite analysis. Results from microsatellite analysis of selected markers for chromosomes 13, 18, 21, and X. AF uncultured amniotic fluid cells, AFc cultured amniotic fluid cells, P placenta tissue, M mother, F father

All tested autosomal markers revealed two distinct alleles with an aberrant ratio of about 2–2.5:1 between the alleles. The ratios for two of the three informative markers on the X chromosome were in the normal range and one exceeded this slightly. No marker for the Y chromosome was detected (Fig. 1 and Additional file 2). We reported a pattern consistent with a triploidy of biparental inheritance comprising one parental haploid set of chromosomes and two further identical haploid chromosome sets of the other parent.

#### Cultured amniotic cells

The autosomal markers revealed for all informative markers two alleles with ratios in the normal range. The ratios for two of three informative markers on the X chromosome exceeded the normal range and one was in the normal range (Fig. 1 and Additional file 2). We reported a normal diploid pattern for the analyzed autosomes and a pattern indicative for trisomy X for the gonosomes.

#### Parents of the fetus

All tested informative markers showed allele ratios in the normal range in both parents. Segregation analyses of the parental alleles enabled us to conclude that the extra haploid set of chromosomes seen on uncultured amniotic cells was of maternal origin. Interestingly, the extra X chromosome observed in the cultured amniotic cells was of paternal origin (paternal isodisomy X).

### Conventional karyotyping

#### Cultured amniotic cells

All 38 metaphases obtained from cultured amniotic cells showed trisomy X; 20 metaphases showed in addition a trisomy 10. None of the metaphases showed triploidy. The karyotype was reported as mos 48,XXX,+10[20]/47,XXX[18].

#### Parents of the fetus

Conventional cytogenetic analyses of the parents revealed normal male (46,XY) and female karyotypes (46,XX).

### FISH analysis

#### Uncultured amniotic cells

FISH analyses for chromosomes 8, 10, 12, 13, 14, 18, 21, X, and Y revealed in 100 evaluated interphase nuclei a mosaicism for one diploid cell line with trisomy X representing 58% of cells and a second diploid cell line with gonosome constellation XX representing 42% of cells. Neither a clone with trisomy 10 nor a clone with triploidy was detected.

#### Cultured amniotic cells

FISH analyses for chromosomes 8, 10, 12, 13, 14, 18, 21, X, and Y revealed in 200 interphase nuclei investigated 94% cells with trisomy X and 37% cells with trisomy 10. For chromosomes 8, 12, 13, 14, 18, and 21, two signals each were detected. No pattern consistent with a triploid clone was observed.

### OncoScan analyses

#### Uncultured amniotic cells

**Fig. 2:**
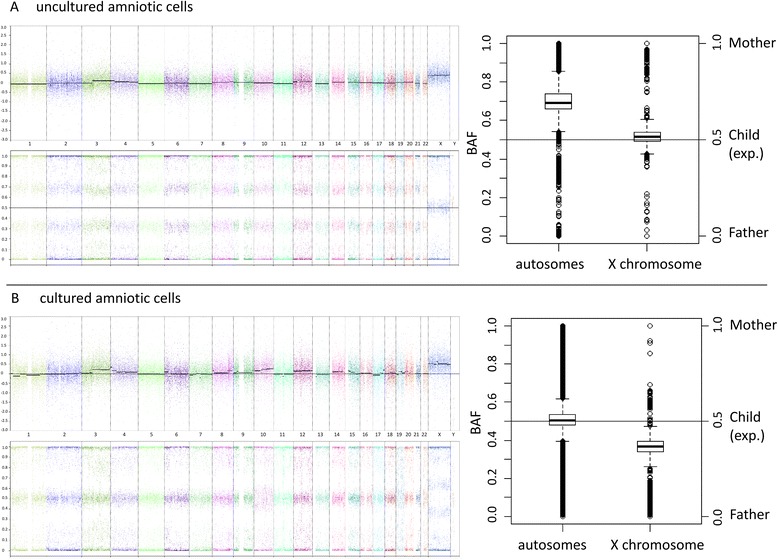
Array (OncoScan) analysis. **a** Results for the uncultured amniotic fluid cells. **b** Results for cultured amniotic fluid cells. The left parts of the figures depict a genome-wide overview of copy number (upper panel. X-axis: chromosomes ordered from 1 to 22, X and Y. Y-axis: copy number state as log2 ratio) and B allele frequency (lower panel. X-axis: chromosome orders from 1 to 22, X and Y. Y-axis: BAF). The right part shows a box plot of the BAF observed in the uncultured (**a**) and cultured (**b**) amniotic fluid cells for the autosomes and for the X chromosome. Only markers for which the parents were informative homozygous (mother BB, father AA or vice versa) were analyzed. The dataset is normalized to a BAF of 1 in the mother and 0 in the father for all analyzed markers. The expected BAF in the analyzed fetal sample is 0.5 for all markers (heterozygous calls). Instead, we observed a skewing of the BAF for the autosomes towards 1 in the uncultured amniotic cells indicating presence of more maternal than paternal alleles. For the X chromosome in the cultured amniotic cells, BAF is shifted towards 0 indicating the extra X chromosome is of paternal origin

A diploid copy number signal was detected for all autosomes. However, the B allele frequencies indicated an unbalanced allele distribution (Fig. 2a). This result confirms the initial QF-PCR result of an excess of maternal alleles over paternal alleles for all tested markers and extends it to all autosomes. For the X chromosome, we observed a diploid pattern for copy number and a balanced B allele frequency (Fig. 2a).

#### Cultured amniotic cells

Copy number indicated diploidy, and the B allele frequencies were balanced for all autosomes except chromosome 10 showing a mosaic copy number gain. For the X chromosome, a copy number gain with the B allele frequency indicating an allele ratio of roughly 2:1 was detected (Fig. 2b).

### Methylation-specific MLPA

#### Uncultured amniotic cells

**Fig. 3:**
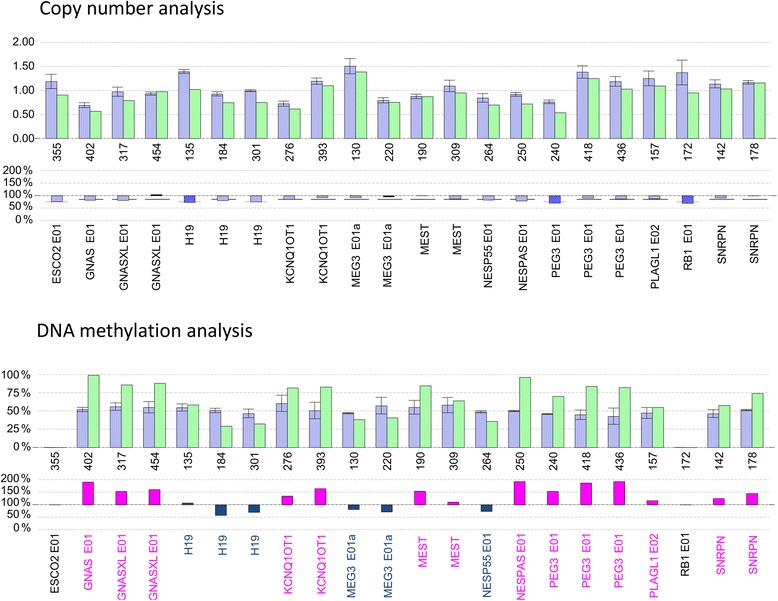
DNA methylation analysis in uncultured amniotic fluid cells. Results of methylation-specific MLPA analysis in uncultured amniotic fluid cells are depicted. Controls consist of three normal samples from amniotic fluid. Upper panel: copy number analysis. Blue bars in the upper histogram: relative control peak area (RPA-C). Green bars in the upper histogram: relative patient peak area (RPA-P). The lower histogram shows the ratio-relative peak area (RPA-P/RPA-C × 100) as blue bars. Lower panel: DNA methylation analysis. Blue bars in the upper histogram: relative control peak area (RPA-C) in % methylation. Green bars in the upper histogram: relative patient peak area (RPA-P) in % methylation. The lower histogram shows the ratio relative peak area (RPA-P/RPA-C × 100): blue bars indicate paternally methylated loci and pink bars indicate maternally methylated loci

We detected DNA methylation disturbances at all methylation-sensitive loci investigated. Thus, we observed a multilocus imprinting disturbance affecting all major regulatory sites for the classical imprinting disorders. The overall DNA methylation pattern was consistent with an enrichment of maternal methylation pattern for all tested regions (Fig. 3 lower panel).

### Analyses of placenta tissue

Pathologic examination of placenta tissue was unremarkable. Chorionic villi were mature with regard to gestational age, organ size was 12 × 9 × 2.5 cm, and weight was 124 g. Thus, the weight of the placenta was regular, lying between the 50^th^ percentile for placental weight of the 18^th^ (105 g) and 19^th^ (125 g) week of gestation. Further genetic analyses of placenta tissue remained inconclusive since we observed significant maternal contamination on microsatellite analyses (Fig. 1 and Additional file 2).

## Discussion

Based on the obtained results of all analyses, we came to the conclusion that the underlying genetic condition in the fetus resulted from a mosaicism for a diploid cell line with genome-wide maternal isodiploidy in combination with a diploid biparental cell line with an extra chromosome X derived from paternal isodisomy X. In culture, predominantly, the latter clone expanded and most likely acquired a trisomy 10 during culturing since this finding was neither confirmed in uncultured amniotic cells nor in an independent culture. Reanalyzing the array raw data with the knowledge of the size of the two detected cell lines from FISH analyses on uncultured amniotic cells, we determined the expected B allele frequency (BAF) based on all informative parental homozygous calls for the autosomes and the X chromosome, respectively. For the autosomes in the uncultured amniotic cells, we expected the normal BAF band of 0.5 to split into two BAF bands at 0.71 and 0.29. Similarly, for the X chromosomes of the cultured amniotic cells, we expected the normal BAF of 0.5 to split at 0.64 and 0.36. These theoretically calculated values were in excellent agreement with the actually observed BAFs of the respective samples (Fig. 2).

We here report on a rare genetic disorder leading to the most extended form of multilocus imprinting disturbance. To the best of our knowledge, up to date, 18 cases of genome-wide parental UPD have been reported in the literature. Sixteen of these refer to patients with mosaic genome-wide UPD of paternal origin [12, 15–20]. Only two patients were reported with clear mosaic maternal genome-wide UPD [12, 21, 22]. In 1995, Strain et al. published the first report of genome-wide maternal UPD in a boy with aggressive behavior, hemifacial hypoplasia, and normal birth weight. They found a cell line with the karyotype 46,XX and genome-wide maternal UPD in nearly all peripheral blood cells and a cell line 46,XY in skin fibroblasts of the patient [21]. The second patient was identified in 2010 in a screening study of patients with SRS-like phenotype. This female patient had a mosaicism of one cell line with genome-wide maternal UPD (46,XX) and a second cell line lacking the second sex chromosome (45,X) [22]. Kotzot et al. list another two reports as mosaic genome-wide maternal UPD [12]. Both studies refer to a mosaic 46,XX/47,XY constellation observed prenatally with the three X chromosomes deriving from the same maternal homologue [23, 24]. However, in both reports, this observation does not extend to the whole chromosome set, and, thus, genome-wide UPD cannot be proven.

The phenotype of genome-wide uniparental diploidies results primarily from functional imbalance of virtually all parentally imprinted loci. Thus, reported symptoms overlap significantly with the characteristic features of imprinting syndromes. Leaving placental-specific imprinted regions aside, ubiquitous imprinted regions in the human were identified on chromosomes 1, 2, 4, 6, 7, 8, 10, 11, 13, 14, 15, 16, 19, 21, and 22 [25]. As mentioned above, some of these chromosomes are related to known imprinting syndromes, but not all of them are associated with specific symptoms when dysregulation affect only single loci. Clinical consequences of maternal UPD for chromosomes 6, 16, and 20 have been investigated recently [26]. Maternal UPD(20) has been proposed as a new imprinting disorder related to prenatal and postnatal growth retardation (IUGR and PNGR), severe feeding difficulties but without characteristic dysmorphisms [27]. Clinical consequences of maternal UPD(6) and UPD(16) are still under debate but most likely do not correspond to a specific phenotype [26]. From these considerations, the clinical phenotype of the patient is expected to consist of mixed features of Silver-Russell syndrome, Temple syndrome, Prader-Willi syndrome, and UPD(20)mat syndrome. At this stage of prenatal development, this would result primarily in intrauterine growth retardation. Indeed, this was one of the leading symptoms observed by prenatal ultrasound. Interestingly, the imprinted region in 11p15.5, harboring the H19/IGF2 IG-DMR and the KCNQ1OT1 TSS-DMR, is known to have a predominant influence on phenotype in patients with MLID [9, 19, 28]. In agreement with this observation, we identified next to intrauterine growth retardation a relative macrocephaly with a ratio of head to abdominal circumference of 1.61 at 18 + 1 weeks of gestation. Moreover, left diaphragmatic hernia (Bochdaleck hernia) and pseudodextrocardia were noted prenatally. These are neither typical symptoms for Silver-Russell syndrome nor for Trisomy X or any of the abovementioned maternal UPDs. In the light of the underlying fundamental genetic disorder, it is likely that the observed malformations are associated with maternal isodiploidy. They could either be associated with disturbed expression of certain imprinted genes or result from unmasking of maternally transmitted recessive mutation(s) in the genome-wide maternal UPD cell line.

Interestingly, some cases with mosaic genome-wide parental UPD contain a mosaicism for a further unbalanced chromosome disorder [18, 22, 29]. This may indicate that cases are missed and interpreted as normal when no second cell line with aberrant copy number of chromosomes is involved. Alternatively, the underlying genetic cause of genome-wide UPD might promote additional chromosome aberrations.

**Fig. 4:**
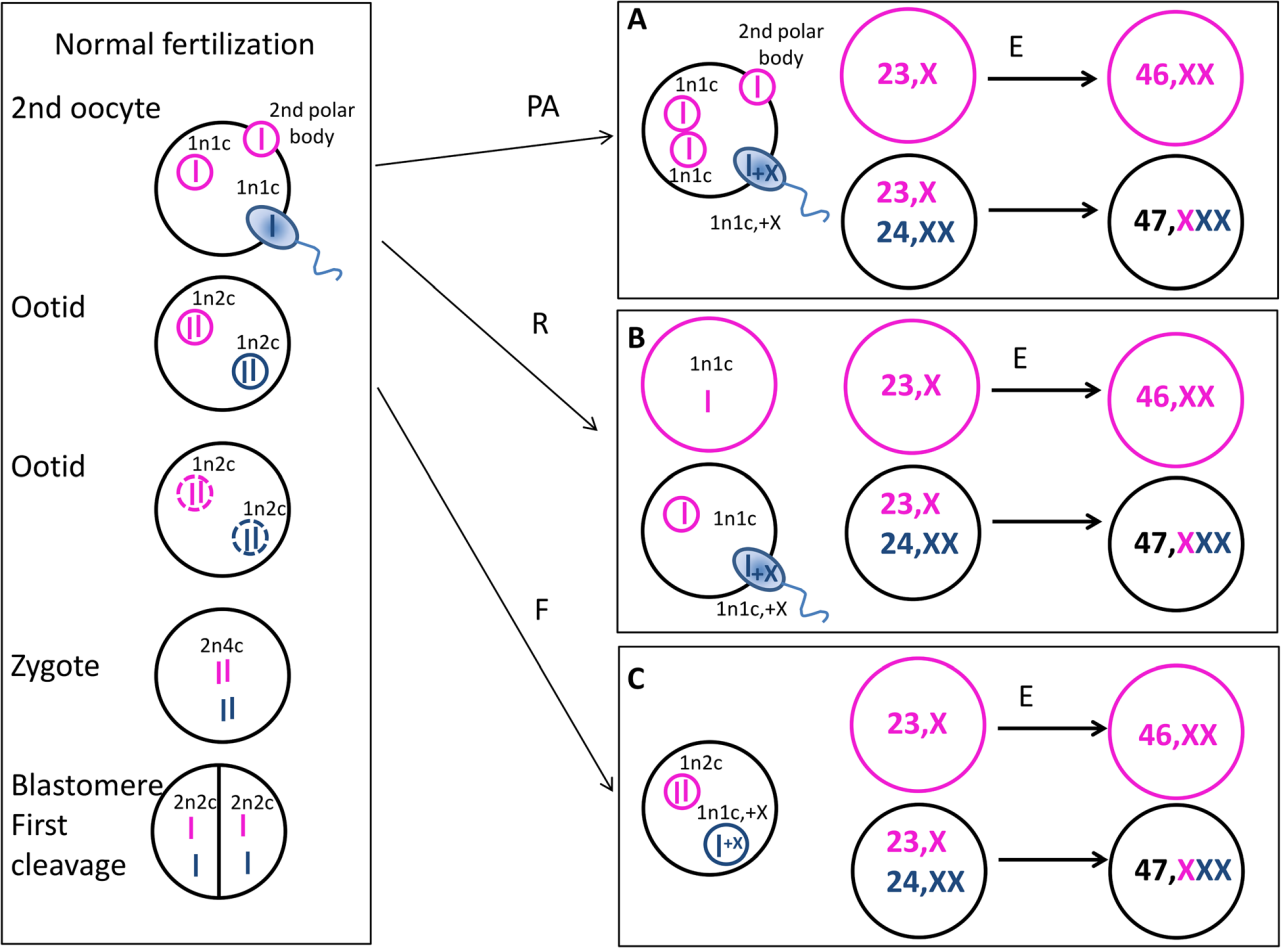
Suggested mechanisms of the observed genetic disorder. **a** Pathogenetic activation (PA) of the maternal pronucleus is assumed as primary event with one pronucleus giving rise to the cell line with genome-wide maternal isodiploidy by endoreplication (E) and mating of the second maternal pronucleus with a sperm bearing two X chromosomes. **b** Retention of the second polar body (R) lead after endoreplication to the origin of the isodiploid cell line. **c** Failure of the paternal pronucleus to duplicate (F) could result in one biparental cell line and a second maternal haploid chromosome set that after endoreplication again could be the origin of the maternal isodiploid cell line. Black circles: biparental inheritance, pink: maternal genetic content, blue: paternal genetic content. n number of chromosome sets, c number of chromatids

The presence of only one maternal allele in both cell lines narrows the time frame of occurrence down from after the first meiotic division to the first cleavage steps. Based on the existing models in similar constellation [16, 22, 29], we propose the possibilities depicted in Fig. 4 for the generation of the isodiploid cell line in combination with the biparental cell line with trisomy X. The scenario depictured in Fig. 4a assumes pathogenetic activation of the maternal pronucleus as primary event with one pronucleus giving rise to the cell line with genome-wide maternal isodiploidy by endoreplication and mating of the second maternal pronucleus with a sperm bearing two X chromosomes. Alternatively, the second polar body may have been restrained and after endoreplication represent the origin of the isodiploid cell line (Fig. 4b). Furthermore, a failure of the paternal pronucleus to duplicate could result in one biparental cell line and a second maternal haploid chromosome set that after endoreplication again could be the origin of the maternal isodiploid cell line (Fig. 4c). For the latter model, the failure of paternal genome duplication needs to be recognized by the cell organism, and as consequence, one maternal haploid chromosome set must get spatially separated from the other chromosomes. While this is possible, it seems to be the least likely mechanism. Whether the extra X chromosome was part of the genetic information in the sperm or was gained later in development of the biparental cell line is impossible to review and, thus, is displayed randomly as being part of the sperm.

Gold standard for investigation of chromosome aberration in prenatal diagnostics remains the conventional karyotype, even though noninvasive tests on free fetal DNA in the maternal blood gain more and more attention. To overcome the need for culturing, rapid investigations for aneuploidies associated with live-born children is routinely performed either by QF-PCR of microsatellite markers or FISH analyses. Choice of methods seems to be distributed rather randomly based on the methodical focus of the individual laboratory with a slight advantage seen in QF-PCR because it can be performed on fewer cells, can detect maternal contamination, and analyses can be automated with many samples processed at the same time [30]. Moreover, QF-PCR is informative regarding parental inheritance while FISH is not. Thus, a genome-wide parental isodiploidy is not detectable by FISH. If we would have performed initial testing for aneuploidies by FISH, we would have reported a mosaicism for a trisomy X, a genetic condition of minor clinical significance often observed as coincidental finding in asymptomatic females. However, QF-PCR does not reflect results on a single cell level, leading to the false interpretation of a triploid chromosome constellation in the presented case. Thus, we would like to alert colleagues based on our experience with this case to the of course well-known but nevertheless challenging limitations of the different methods applied in routine prenatal genetic testing.

## Conclusions

We here report the third case of mosaic genome-wide maternal UPD leading to the most extended form of multilocus imprinting disturbance. Our findings highlight the technical challenges to come to the correct diagnosis in a routine prenatal setting, which is only possible by a combination of analysis of copy number per cell and parental inheritance to unmask genome-wide UPD. In line with previous studies, we observed mosaicism for a second cell line with an unbalanced chromosome disorder, namely a cell line of biparental inheritance with trisomy X and paternal isodisomy X. This case adds more data on clinical outcome of genome-wide maternal UPD, but further reports are needed to draw clear genotype-phenotype correlations.

## Additional files

Additional file 1: Primer sequences applied for quantitative fluorescence-polymerase chain reaction (QF-PCR) for chromosomes 13, 18, 21, X, and Y. F: forward primer; R: reverse primer. (PDF 31 kb)

Additional file 2: Results from quantitative fluorescence-polymerase chain reaction (QF-PCR) for chromosomes 13, 18, 21, X, and Y including peak areas and allele ratios. AF: uncultured amniotic fluid cells; AFc: cultured amniotic fluid cells; P: placenta tissue; M: mother, F: father, n.a. not applicable. (PDF 79 kb)

## Abbreviations

AS: Angelman syndrome
BAF: B allele frequency
BWS: Beckwith-Wiedemann syndrome
FISH: Fluorescence in situ hybridization
KOS14: Kagami-Ogata syndrome
MIP: Molecular inversion probe
MLID: Multilocus imprinting disturbance
PHA: Phytohaemagglutinin
PHP-IB: Pseudo-hypoparathyreoidism type IB
PWS: Prader-Willi syndrome
QF-PCR: Quantitative Fluorescence-Polymerase Chain Reaction
SRS: Silver-Russell syndrome
TNDM: Transient neonatal diabetes mellitus
TS14: Temple syndrome
UPD: Uniparental disomy
UPDmat: Maternal uniparental disomy
UPDpat: Paternal uniparental disomy
