# Supplementary material for: Mosaic genome-wide maternal isodiploidy: an extreme form of imprinting disorder presenting as prenatal diagnostic challenge
Source: Clin Epigenetics. 2017 Oct 13;9:111. doi: 10.1186/s13148-017-0410-y (PMC5640928; doi:10.1186/s13148-017-0410-y)
Supplement: Supplementary file 1 — Primer sequences applied for quantitative fluorescence-polymerase chain reaction (QF-PCR) for chromosomes 13, 18, 21, X, and Y. F: forward primer; R: reverse primer. (PDF 31 kb) [file 13148_2017_410_MOESM1_ESM.pdf]

Marker	Primer sequence 5' → 3'	label	chromosome
D13S742.F	ATAACTGGGCTAGGAATGGAAATA	HEX	13
D13S742.R	GACTTCCCAATTCAGGAGGACT		13
D13S634.F	GGCAGATTCAATAGGATAAATAGA	6-FAM	13
D13S634.R	GTAACCCCTCAGGTTCTCAAGTCT		13
D13S628.F	TAACATTCATTGTCCCTTACAGAT	NED	13
D13S628.R	GCAAGGCTATCTAACGATAATTCA		13
D18S391.F	GGACTTACCACAGGCAATGTGACT	HEX	18
D18S391.R	TAGACTTCACTATTCCCATCTGAG		18
D18S386.F	TGAGTCAGGAGAATCACTTGGAAC	HEX	18
D18S386.R	CTCTTCCATGAAGTAGCTAAGCAG		18
D18S1002.F	CAAAGAGTGAATGCTGTACAAACAGC	6-FAM	18
D18S1002.R	TGACTCAATGGATAAACAGATAT		18
D18S535.F	CAGCAAACTTCATGTGACAAAAGC	6-FAM	18
D18S535.R	CAATGGTAACCTACTATTTACGTC		18
D21S11.F	TTTCTCAGTCTCCATAAATATGTG	6-FAM	21
D21S11.R	GATGTTGTATTAGTCAATGTTCTC		21
D21S1270.F	CTATCCCACTGTATTATTCAGGGC	6-FAM	21
D21S1270.R	TGAGTCTCCAGGTTGCAGGTGACA		21
IFNAR.F	GTTCTTCATTTGATCTTAGCCATC	HEX	21
IFNAR.R	GTGAGATAACTGGCAAGAAGATAA		21
D21S1437.F	ATGTACATGTGTCTGGGAAGG	6-FAM	21
D21S1437.R	TTCTCTACATATTTACTGCCAACA		21
PentaD.F	GAAGGTCGAAGCTGAAGTG	6-FAM	21
PentaD.R	ATTAGAATTCTTTAATCTGGACACAAG		21
D21S1446.F	ATGTACGATACGTAATACTTGACAA	6-FAM	21
D21S1446.R	GTCCCAAAGGACCTGCTC		21
AMX/Y.F	CCCTGGGCTCTGTAAAGAATAGTG	NED	X/Y
AMX/Y.R	ATCAGAGCTTAAACTGGGAAGCTG		X/Y
HPRT.F	ATGCCACAGATAATACACATCCCC	6-FAM	X
HPRT.R	CTCTCCAGAATAGTTAGATGTAGG		X
DXS1283E.F	AGTTTAGGAGATTATCAAGCTG	HEX	X
DXS1283E.R	TCAAAGTGATCGACAACTACTCAGA		X
DXS981.F	CTCCTTGTGGCCTTCCTTAAATG	6-FAM	X
DXS981.R	TTCTCTCCACTTTTCAGAGTCA		X
DXS6854.F	AGCACTTCTCCTACAACCCTC	6-FAM	X
DXS6854.R	CAGCCTGGGCAGTAGAGACT		X
P39.F	AGCACATGGTATAATGAACCTCCACG	6-FAM	X
P39.R	CAGTGTGAGTAGCATGCTAGCATTTG		X
