# Supplementary material for: Mosaic genome-wide maternal isodiploidy: an extreme form of imprinting disorder presenting as prenatal diagnostic challenge
Source: Clin Epigenetics. 2017 Oct 13;9:111. doi: 10.1186/s13148-017-0410-y (PMC5640928; doi:10.1186/s13148-017-0410-y)
Supplement: Supplementary file 2 — Results from quantitative fluorescence-polymerase chain reaction (QF-PCR) for chromosomes 13, 18, 21, X, and Y including peak areas and allele ratios. AF: uncultured amniotic fluid cells; AFc: cultured amniotic fluid cells; P: placenta tissue; M: mother, F: father, n.a. not applicable. (PDF 79 kb) [file 13148_2017_410_MOESM2_ESM.pdf]

Sample	Marker	Size 1	Size 2	Size 3	Peak Area 1	Peak Area 2	Peak Area 3	Bigger Peak Area/ Smaller Peak Area (allele ratio)
AF	AMX_Y	104,24			98735,0			n.a.
AF	D13S628	431,79			83692,0			n.a.
AF	D13S634	400,59	411,97		18333,0	37545,0		2,05
AF	D13S742	267,86	276,8		31711,0	66728,0		2,10
AF	D18S1002	170,66	174,67		45145,0	17724,0		2,55
AF	D18S386	360,26	377,81		14919,0	32485,0		2,18
AF	D18S391	151,01	155,25		49214,0	104668,0		2,13
AF	D18S535	479,44	483,44		17434,0	37077,0		2,13
AF	D21S11	258,46	266,13		12282,0	27670,0		2,25
AF	D21S1270	300,71	317,39		21479,0	7444,0		2,89
AF	D21S1437	110,4	126,33		27218,0	61461,0		2,26
AF	D21S1446	220,46	222,66		59972,0	24082,0		2,49
AF	PENTA D	409,68			34758,0			n.a.
AF	IFNAR	474,69	486,88		10796,0	19132,0		1,77
AF	DXS1283E	306,26	312,64		25723,0	14801,0		1,74
AF	DXS6854	94,81	106,65		88140,0	111757,0		1,27
AF	DXS981	249,05	250,00		104857,0	80906,0		1,30
AF	HPRT	282,02			96778,0			n.a.
AF	P39	152,39			108839,0			n.a.
AFc	AMX_Y	104,3			26696,0			n.a.
AFc	D13S628	431,76			49026,0			n.a.
AFc	D13S634	400,6	412,07		18333,0	16878,0		1,09
AFc	D13S742	267,79	276,82		20760,0	24595,0		1,18
AFc	D18S1002	170,68	174,73		16435,0	14673,0		1,12
AFc	D18S386	360,46	378		13376,0	13274,0		1,01
AFc	D18S391	151,34	155,43		22508,0	19652,0		1,15
AFc	D18S535	479,58	483,54		22414,0	18178,0		1,23
AFc	D21S11	258,2	265,85		15072,0	18650,0		1,24
AFc	D21S1270	300,81	317,25		10165,0	8409,0		1,21
AFc	D21S1437	110,47	126,32		15374,0	12892,0		1,19
AFc	D21S1446	220,59	222,73		14737,0	13681,0		1,08
AFc	PENTA D	409,61			22863,0			n.a.
AFc	IFNAR	475,07	487,07		14693,0	13587,0		1,08
AFc	DXS1283E	306,14	312,52		20106,0	24415,0		1,21
AFc	DXS6854	95,15	106,92		99279,0	53733,0		1,85
AFc	DXS981	248,95	249,88		54460,0	109212,0		2,01
AFc	HPRT	282,03			42490,0			n.a.
AFc	P39	152,68			91791,0			n.a.
P	AMX_Y	104,38			128253,0			maternal contamination
P	D13S628	431,85	459,41		50523,0	3652,0		maternal contamination
P	D13S634	400,59	408,11	412,01	15065,0	4827,0	17954,0	maternal contamination
P	D13S742	257,04	267,9	276,92	8375,0	25291,0	30688,0	maternal contamination
P	D18S1002	170,77	174,7	182,63	34061,0	22296,0	5505,0	maternal contamination
P	D18S386	360,43	370,59	377,98	8245,0	1775,0	8740,0	maternal contamination
P	D18S391	151,11	155,36	159,53	73740,0	92884,0	16694,0	maternal contamination
P	D18S535	479,41	483,42		13860,0	13459,0		maternal contamination
P	D21S11	258,54	266,2		10071,0	14609,0		maternal contamination
P	D21S1270	300,69	317,37	321,54	11008,0	6414,0	1520,0	maternal contamination
P	D21S1437	110,39	126,27		58957,0	90619,0		maternal contamination
P	D21S1446	220,6	222,77		90022,0	52644,0		maternal contamination
P	IFNAR	474,88	478,81	486,87	4713,0	1592,0	5442,0	maternal contamination
P	DXS1283E	306,19	310,51	312,62	27546,0	23908,0	22653,0	maternal contamination
P	DXS6854	94,9	106,67	110,61	120188,0	78250,0	14440,0	maternal contamination
P	DXS981	248,94	249,88	252,92	97260,0	163549,0	16548,0	maternal contamination
P	HPRT	281,98	289,6		180108,0	12865,0		maternal contamination
P	P39	152,34	158,35		168650,0	9961,0		maternal contamination
M	AMX_Y	104,28			135213,0			n.a.
M	D13S628	431,89	459,41		97802,0	77425,0		1,26
M	D13S634	408,16	411,95		57516,0	50675,0		1,13
M	D13S742	256,87	276,77		105979,0	90563,0		1,17
M	D18S1002	170,59	182,52		49922,0	44555,0		1,12
M	D18S386	370,67	377,97		49890,0	45128,0		1,11
M	D18S391	155,25	159,41		122350,0	121596,0		1,01
M	D18S535	479,42	483,42		62978,0	55553,0		1,13
M	D21S11	265,98			61104,0			n.a.
M	D21S1270	300,69	321,35		37113,0	28355,0		1,31
M	D21S1437	126,37			149550,0			n.a.
M	D21S1446	220,47			156771,0			n.a.
M	PENTA D	409,74	414,52		49777,0	49533,0		1,00
M	IFNAR	478,82	487		55216,0	49484,0		1,12
M	DXS1283E	306,19	310,4		58704,0	34344,0		1,71
M	DXS6854	106,75	110,66		139802,0	135146,0		1,03

M	DXS981	248,93	252,83		148687,0	124841,0		1,19
M	HPRT	281,98	289,6		93973,0	87009,0		1,08
M	P39	152,36	158,33		98706,0	73962,0		1,33
F	AMX_Y	104,25	109,61		64937,0	61498,0		1,06
F	D13S628	431,85			141273,0			n.a.
F	D13S634	400,59	408,18		44624,0	42923,0		1,04
F	D13S742	267,79			131915,0			n.a.
F	D18S1002	174,65	178,59		38156,0	37745,0		1,01
F	D18S386	355,71	360,37		38629,0	34944,0		1,11
F	D18S391	151,11	159,41		88489,0	87020,0		1,02
F	D18S535	479,35			97441,0			n.a.
F	D21S11	258,37	262,23		28966,0	26348,0		1,10
F	D21S1270	317,26			45190,0			n.a.
F	D21S1437	110,39	138,74		73104,0	58478,0		1,25
F	D21S1446	216,35	222,72		72892,0	76360,0		1,05
F	PENTA D	400	409,72		44690,0	46841,0		1,05
F	IFNAR	474,8	478,74		36924,0	35665,0		1,04
F	DXS1283E	312,47			43181,0			n.a.
F	DXS6854	94,87			152135,0			n.a.
F	DXS981	249,76			138229,0			n.a.
F	HPRT	281,95			93145,0			n.a.
F	P39	152,46			100827,0			n.a.
